# Echinochrome A Protects Mitochondrial Function in Cardiomyocytes against Cardiotoxic Drugs

**DOI:** 10.3390/md12052922

**Published:** 2014-05-13

**Authors:** Seung Hun Jeong, Hyoung Kyu Kim, In-Sung Song, Seon Joong Lee, Kyung Soo Ko, Byoung Doo Rhee, Nari Kim, Natalia P. Mishchenko, Sergey A. Fedoryev, Valentin A. Stonik, Jin Han

**Affiliations:** 1 National Research Laboratory for Mitochondrial Signaling, Department of Physiology, College of Medicine, Cardiovascular and Metabolic Disease Center (CMDC), Inje University, Busan 614-735, Korea; E-Mails: shjeong96@gmail.com (S.H.J.); estrus74@gmail.com (H.K.K.); microvirus@hanmail.net (I.-S.S.); jesusyi79@naver.com (S.J.L.); kskomd@paik.ac.kr (K.S.K.); bdrhee@hanmail.net (B.D.R.); narikim43@gmail.com (N.K.); 2 Department of Health Sciences and Technology, Graduate School of Inje University, Busan 614-735, Korea; 3 G.B. Elyakov Pacific Institute of Bioorganic Chemistry, Far-Eastern Branch of the Russian Academy of Science, Prospect 100 let Vladivostoku, 159, Vladivostok 690022, Russia; E-Mails: mischenkonp@mail.ru (N.P.M.); fedoreev-s@mail.ru (S.A.F.); stonik@piboc.dvo.ru (V.A.S.)

**Keywords:** echinochrome A, mitochondrial function, cardiotoxic drugs, SNP, tBHP, doxorubicin

## Abstract

Echinochrome A (Ech A) is a naphthoquinoid pigment from sea urchins that possesses antioxidant, antimicrobial, anti-inflammatory and chelating abilities. Although Ech A is the active substance in the ophthalmic and cardiac drug Histochrome^®^, its underlying cardioprotective mechanisms are not well understood. In this study, we investigated the protective role of Ech A against toxic agents that induce death of rat cardiac myoblast H9c2 cells and isolated rat cardiomyocytes. We found that the cardiotoxic agents *tert*-Butyl hydroperoxide (tBHP, organic reactive oxygen species (ROS) inducer), sodium nitroprusside (SNP; anti-hypertension drug), and doxorubicin (anti-cancer drug) caused mitochondrial dysfunction such as increased ROS level and decreased mitochondrial membrane potential. Co-treatment with Ech A, however, prevented this decrease in membrane potential and increase in ROS level. Co-treatment of Ech A also reduced the effects of these cardiotoxic agents on mitochondrial oxidative phosphorylation and adenosine triphosphate level. These findings indicate the therapeutic potential of Ech A for reducing cardiotoxic agent-induced damage.

## 1. Introduction

**Figure 1:**
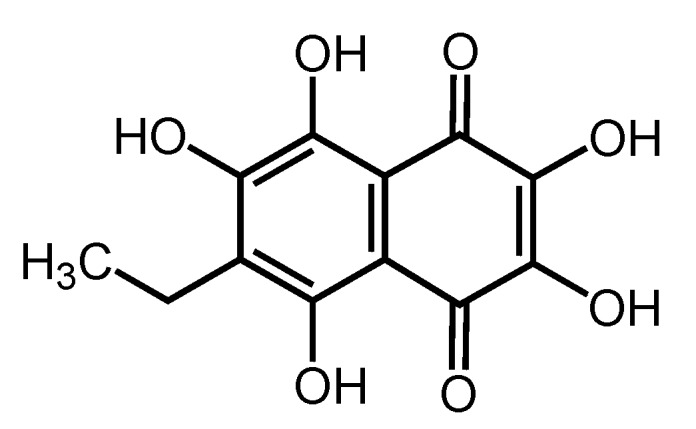
Chemical structure of echinochrome A (Ech A).

Echinochrome A (Ech A) is the most common dark-red pigment in sea urchin shells and spines [1] and is also found in eggs, larvae, and different tissues of adult sea urchins [2]. Chemically, Ech A is known as 6-ethyl-2,3,5,7,8-pentahydroxy-1,4-naphthoquinone (Figure 1). Ech A is a water-insoluble compound that possesses strong antioxidant effects and is the active substance (P N002362/01) in the drug Histochrome. One form of Histochrome is used in the treatment of ocular diseases [3,4]. In Russia, Histochrome is often used in ophthalmic practice to treat intraocular hemorrhage, diabetic retinopathy, dystrophies, central retinal vein thrombosis, and post-traumatic hemorrhage [5,6]. Another form of Histochrome is used for preventing reperfusion damage during myocardial infarction [7,8]. Although protection against ischemic reperfusion in myocardial infarction and injury is realized via reactive oxygen species (ROS) scavenging and ion chelating [9,10,11], the cardioprotective mechanism of Ech A is not well understood.

Several chemotherapeutic drugs, such as sodium nitroprusside (disodium nitroferricyanide, SNP) and doxorubicin (Dox), can exert genotoxic effects on adjacent normal cells due to oxidative stress, thereby leading to secondary malignancies. SNP is a clinically effective hypertensive drug used in pediatric intensive care units for acute heart failure [12]. Low doses of SNP have protective effects against cell injury via iron ion chelation [13], but high doses of SNP inhibit mitochondrial cytochrome C oxidase by liberating cyanide ions, which induces severe cardiac toxicity [14,15]. Dox is an anthracycline anti-cancer drug that is widely used to treat solid tumors and hematological malignancies via the inhibition of topoisomerase and the subsequent blockade of DNA resealing during replication [16]. However, Dox is toxic to both tumor cell and healthy tissues [17]. Thus, the clinical application of these drugs is limited due to their serious cardiotoxicity, which results in the permanent loss of cardiomyocytes [18]. Numerous studies suggest that SNP [19] and Dox [20,21,22], as well as *tert*-Butyl hydroperoxide (tBHP), induce the production of ROS in mitochondria [23]. Importantly, ROS is a known cause of cardiomyopathy, which involves dysfunctional and morphological changes in heart tissue [24].

In the present study, we investigated the potential protective effect of Ech A against the cardiotoxic drugs tBHP, SNP, and Dox, which induce the death of H9c2 cells and isolated rat cardiomyocytes. Furthermore, we tested whether co-treatment with Ech A prevents the adverse effects of these cardiotoxic drugs on mitochondrial functions (e.g., oxygen consumption, adenosine triphosphate (ATP) production capacity, ROS homeostasis).

## 2. Results and Discussion

### 2.1. Ech A Inhibited Cardiotoxic Agent-Induced Cell Death

**Figure 2:**
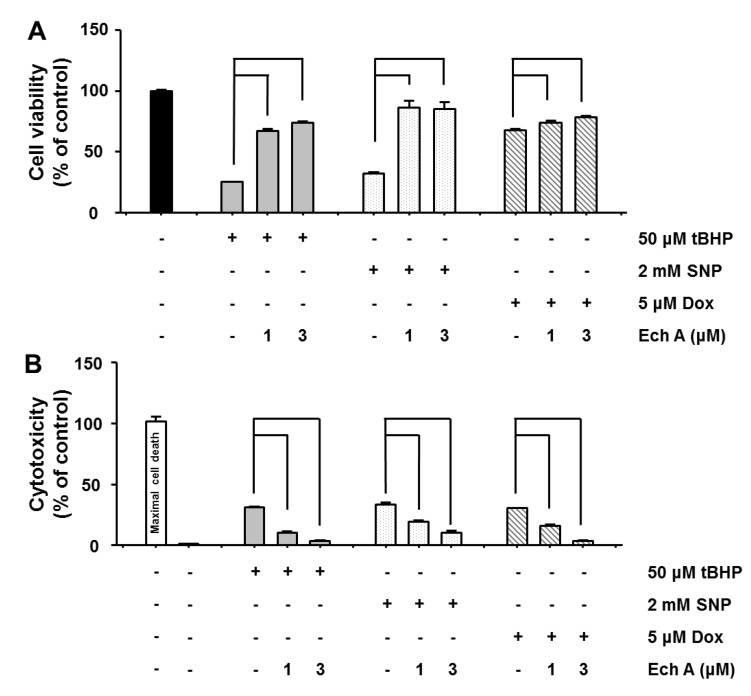
Ech A inhibited cardiotoxic agent-induced cell death. (**A**) Rat cardiac myoblast H9c2 cells were treated with cardiotoxic agents (50 μM tBHP, 2mM SNP, or 50 μM tBHP) for 24 h, and cell survival rates were measured using an MTT assay. Cardiotoxic agents significantly reduced cell survival rate, but co-treatment with Ech A (1 or 3 μM) significantly prevented this drug-induced cell death. (**B**) In addition, cardiotoxic agents significantly induced cytotoxicity, but co-treatment with Ech A significantly prevented cardiotoxicity agent-induced cytotoxicity. Positive control was maximal cell death which was indicated by cells treated with cell lysis solution (digitonin solution). Negative control was untreated cells. Four independent *in vitro* experiments were performed. *P* < 0.05 *vs*. cardiotoxic agent single treatment group*.*

Cardiotoxic doses of tBHP, SNP, and Dox were based on previous studies [25,26,27]. To confirm the effectiveness of these doses, rat cardiac myoblast H9c2 cells were treated with 50 μM tBHP, 2 mM SNP, or 5 μM Dox for 24 h. We found that all cardiotoxic agents reduced H9c2 cell viability (tBHP: 25.0% ± 0.4%, SNP: 32.0% ± 1.0%, Dox: 67.0% ± 0.8%). Next, we investigated whether Ech A reduces cardiotoxic agent-induced cell death by treating H9c2 cells with tBHP, SNP, or Dox in the presence of 0, 1, or 3 μM Ech A for 24 h. We found that co-treatment with Ech A significantly prevented the cell death caused by tBHP, SNP, and Dox (Figure 2A). To confirm cell viability, we tested cytotoxicity assay. Likewise, Ech A significantly reduced cardiotoxic agent-induced cytotoxicity (Figure 2B). These results indicate that Ech A could protect cells from death induced by direct oxidative stress, suggesting its potential use in combined therapy to reduce the adverse effects of cardiotoxic agents.

### 2.2. Ech A Attenuated Cardiotoxic Agent-Induced Mitochondrial Damage

**Figure 3:**
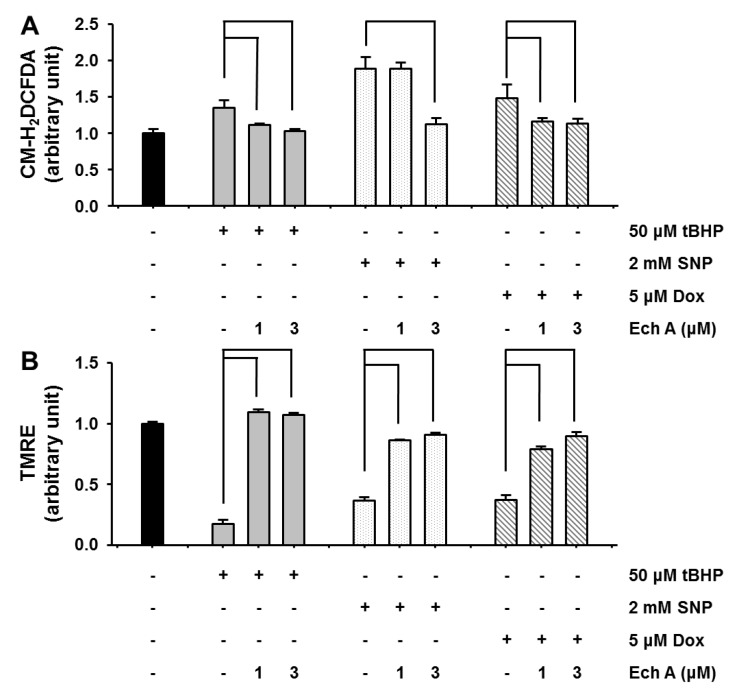
Ech A attenuated cardiotoxic agent-induced mitochondrial damage in H9c2 cells. Cells were treated with cardiotoxic agents for 1 h, after which ROS level and mitochondrial membrane potential were measured using a microplate assay. (**A**) Cardiotoxic agents rapidly increased ROS (CM-H_2_DCFDA) level and (**B**) decreased mitochondrial membrane potential (ΔΨm, TMRE). Co-treatment with Ech A attenuated the increase in reactive oxygen species (ROS) level and preserved mitochondrial membrane potential. Four independent *in vitro* experiments were performed. *P* < 0.05 *vs*. cardiotoxic agent single treatment group*.*

To test whether Ech A could protect mitochondria against cardiotoxic agents, we examined cardiotoxic agent-induced alterations in ROS level and mitochondrial membrane potential in the presence of 1 or 3 μM Ech A in H9c2 cells. After 1-h treatment with cardiotoxic agents, we observed a depolarization of mitochondrial membrane potential and an increase in ROS production. Co-treatment with Ech A, however, significantly prevented the increase in ROS level (Figure 3A) and recovered mitochondrial membrane potential depolarization (Figure 3B).

**Figure 4:**
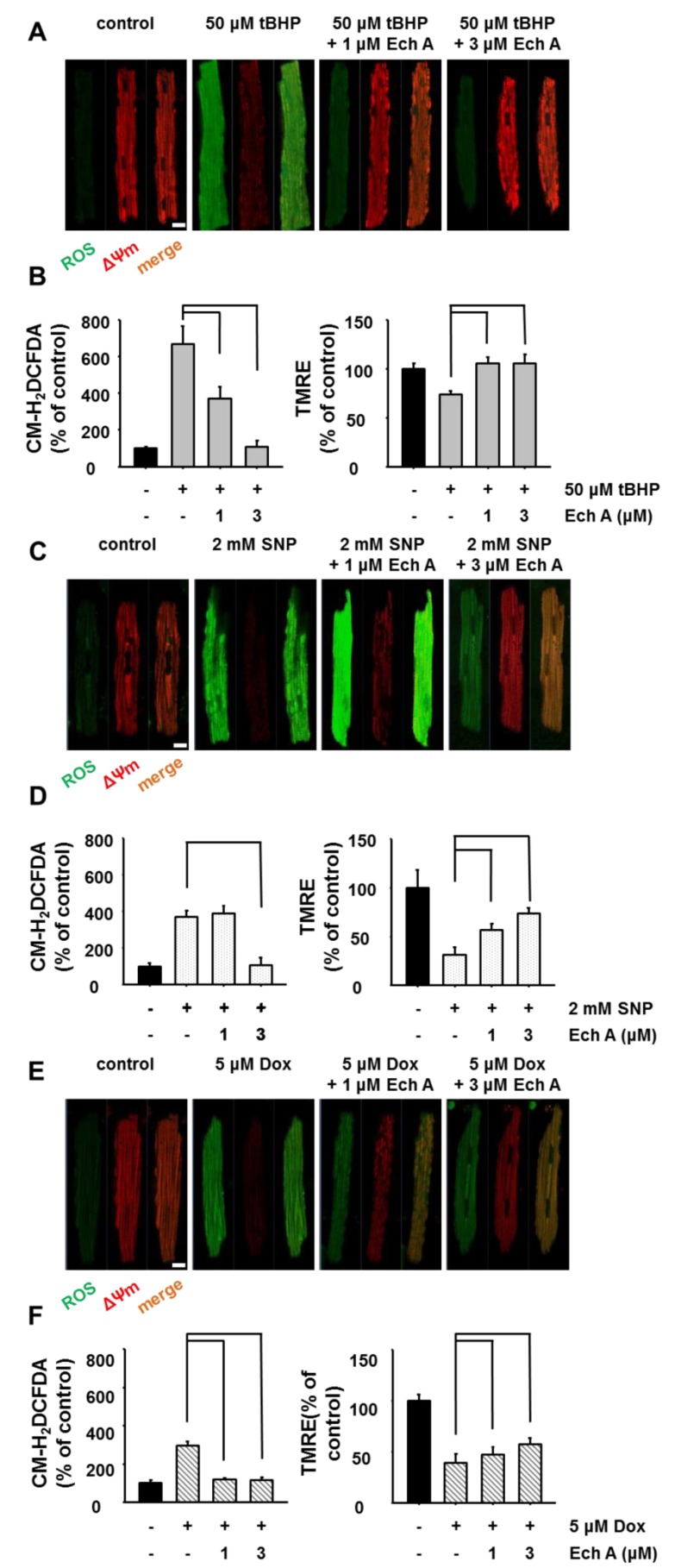
Ech A attenuated cardiotoxic agent-induced mitochondrial damage in isolated rat cardiomyocytes. Isolated rat cardiomyocytes were treated with cardiotoxic agents for 1 h, after which ROS level (CM-H_2_DCFDA, green color) and mitochondrial membrane potential (ΔΨm, TMRE, red color) were measured using confocal microscopy. (**A**) Cardiotoxic agent, *tert*-Butyl hydroperoxide (tBHP) rapidly increased ROS level (green, ROS indicator) and decreased mitochondrial membrane potential (red, ΔΨm). Co-treatment with Ech A attenuated the increase in ROS level and preserved mitochondrial membrane potential. Data are summarized in (**B**). (**C**) Sodium nitroprusside (SNP) rapidly increased ROS level (green, ROS indicator) and decreased mitochondrial membrane potential (red, ΔΨm). Co-treatment with Ech A attenuated the increase in ROS level and preserved mitochondrial membrane potential. Data are summarized in (**D**). (**E**) Dox rapidly increased ROS level (green, ROS indicator) and decreased mitochondrial membrane potential (red, ΔΨm). Co-treatment with Ech A attenuated the increase in ROS level and preserved mitochondrial membrane potential. Data are summarized in (**F**). Four independent *in vitro* experiments were performed. *P* < 0.05 *vs*. cardiotoxic agent single treatment group*.* Scale bar = 20 μm.

Since established cell line can have different responses to drugs compared to intact mammalian cells, we next confirmed the cardioprotective effect of Ech A in freshly isolated rat cardiomyocytes. We found that co-treatment with Ech A (1 or 3 μM) attenuated the cardiotoxic agent-induced increase in ROS level (Figure 4A,C,E; green: CM-H_2_DCFDA) and depolarization of mitochondrial membrane potential (Figure 4A,C,E; red: TMRE).

**Figure 5:**
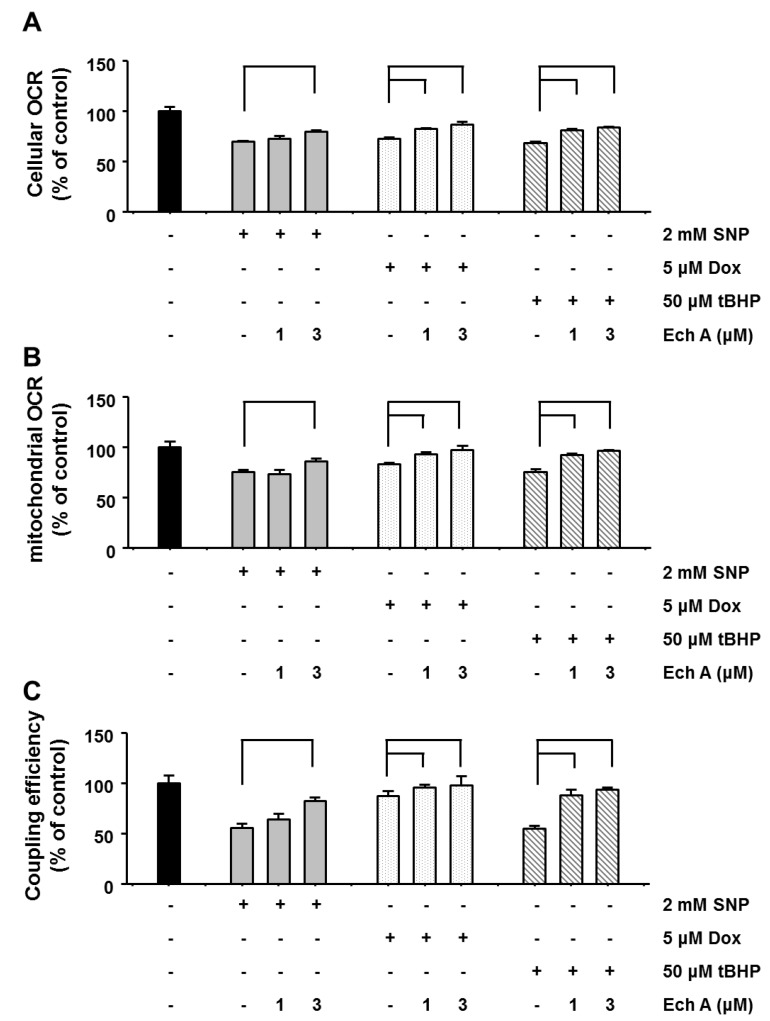
Ech A protected mitochondrial functions against cardiotoxic agent-induced damage. H9c2 cells were treated with cardiotoxic agents for 1 h cellular and mitochondrial oxygen consumption rate (OCR) and coupling efficiency was measured using an XF24 analyzer, respectively. (**A**) Cardiotoxic agents inhibited cellular OCR, but co-treatment with Ech A prevented this inhibition. (**B**) Cardiotoxic agents inhibited mitochondrial OCR, but co-treatment with Ech A prevented this inhibition. (**C**) Moreover, Cardiotoxic agents also inhibited coupling efficiency, but co-treatment with Ech A prevented this inhibition. Four independent *in vitro* experiments were performed. *P* < 0.05 *vs*. cardiotoxic agent single treatment group*.*

**Figure 6:**
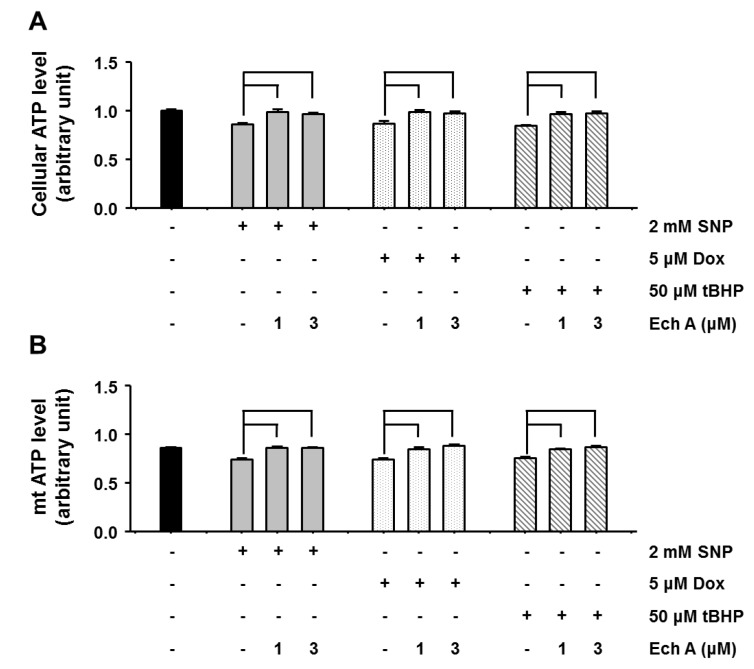
Ech A protected mitochondrial functions against cardiotoxic agent-induced damage. H9c2 cells were treated with cardiotoxic agents for 1 h. Cellular and mitochondrial ATP level were measured using the mitochondrial ToxGlo assay, respectively. (**A**) Cardiotoxic agents reduced cellular ATP, but co-treatment with Ech A prevented this reduction. (**B**) Moreover, cardiotoxic agents also reduced mitochondrial ATP, but co-treatment with Ech A prevented this reduction. Four independent *in vitro* experiments were performed. *P* < 0.05 *vs*. cardiotoxic agent single treatment group*.*

We further examined whether Ech A preserves mitochondrial energy production capacity in the presence of cardiotoxic agents. Mitochondrial metabolic function was determined by measuring mitochondrial oxygen consumption rate (OCR) using an XF24 analyzer. Treatment with cardiotoxic agents significantly decreased cellular and mitochondrial OCR, but co-treatment with Ech A preserved cellular and mitochondrial OCR in treated cells (Figure 5A,B). Moreover, treatment with cardiotoxic agents significantly decreased mitochondrial coupling efficiency but co-treatment with Ech A preserved coupling efficiency in treated cells (Figure 5C). Coupling efficiency indicated ATP synthetics ability by mitochondrial oxidative phosphorylation. We measured cellular and mitochondrial ATP level whether treatment with Ech A preserved mitochondrial function from cardiotoxic agent treatment. Cardiotoxic agents significantly decreased cellular and mitochondrial ATP level, whereas Ech A treatment significantly prevented this drug-induced decline (Figure 6A,B). These results suggest that Ech A has the potential to protect mitochondria against various mitotoxic drugs or stimuli through preventing ROS generation and mitochondrial membrane potential depolarization.

### 2.3. Ech A Regulated ERK1/2, JNK, and p38 Signaling Pathways

**Figure 7:**
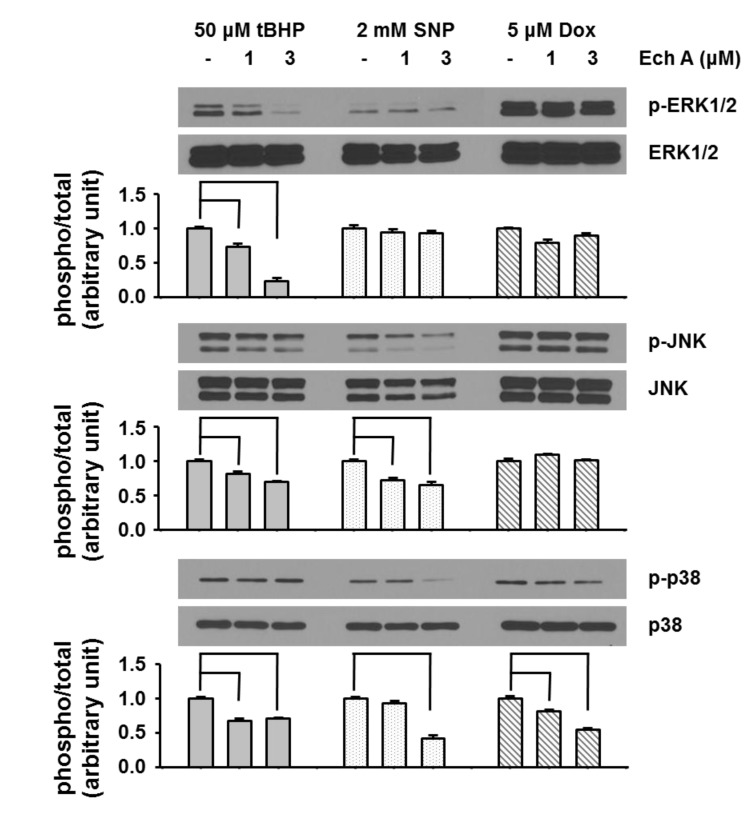
Ech A regulates ERK1/2, JNK, and p38 signaling pathways. H9c2 cells were treated with cardiotoxic agents for 1 h, after which western blotting was performed. And we analyzed the western blot bands though quantification with multi-gage. Cardiotoxic agents induced phosphorylation of ERK1/2, JNK, and p38, but co-treatment with Ech A inhibited this phosphorylation. Three independent *in vitro* experiments were performed. *P* < 0.05 *vs*. cardiotoxic agent single treatment group.

Mitogen-activated protein kinases (MAPKs, including EKR1/2, JNK, and p38) are members of the serine/threonine protein kinase family that are widely distributed in eukaryotic cells and involved in various cellular functions, such as cell growth, motility, and cell death [13,28,29]. Several studies suggest that cardiotoxic agents activate MAPKs [30,31]. The downstream targets of the MAPK pathway include several transcription factors whose activities are enhanced by phosphorylation, and the selective activation of the MAPK pathway can lead to cell death. Using western blot analysis, we determined whether cardiotoxic agents induce MAPK pathway activation and whether Ech A co-treatment reduces this activation. Similar to our findings, previous studies also report that tBHP [32,33,34], SNP [35], and Dox [36,37] induce phosphorylation of ERK1/2, JNK, and p38. At the current survey we found, however, that co-treatment with Ech A attenuated this phosphorylation (Figure 7). Specifically, co-treatment of Ech A with tBHP prevented the phosphorylation of ERK1/2 and JNK. On the other hand, co-treatment of Ech A with SNP prevented the phosphorylation of JNK and p38, while co-treatment of Ech A with Dox prevented the phosphorylation of p38.

## 3. Experimental Section

### 3.1. Chemicals

tBHP (t-BuOOH), SNP (disodium nitroferricyanide), and Dox were obtained from Sigma-Aldrich (St. Louis, MO, USA). Ech A (6-ethyl-2,3,5,7,8-pentahydroxy-1,4-naphthoquinone) was isolated from the sea urchin *Scaphechinus mirabilis* (Agassiz) using a previously described method [38]. The purity of Ech A was >99% according to liquid chromatography-mass spectrometry (LS-MS) data (Shimadzu LCMS-2020, Kyoto, Japan).

### 3.2. Cell Culture

Rat cardiac myoblast H9c2 cells (American Type Culture Collection, Manassas, VA, USA) were maintained in Dulbecco’s Modified Eagle Medium (DMEM) supplemented with 10% heat-inactivated fetal bovine serum, 50 U/mL penicillin, and 50 μg/mL streptomycin (Lonza, Walkersville, MD, USA).

#### 3.2.1. Measurement of Cell Viability

H9c2 cells were cultured at 2 × 10^4^ cells/well in 96-well tissue culture plates. After 16 h, cells were treated with 50 μM tBHP, 2 mM SNP, or 5 μM Dox in the presence of 0, 1, or 3 μM Ech A for 24 h. Cell viability was assessed by quantitative colorimetric assay with MTT (3-(4,5-dimethylthiazol-2-yl)-2,5-diphenyltetrazolium bromide; Sigma-Aldrich, St. Louis, MO, USA). The extent of MTT transformation into formazan, an index of the mitochondrial activity of living cells, was quantified by measuring optical density at 570 nm using a microplate reader (Molecular Device, Sunnyvale, CA, USA).

#### 3.2.2. Measurement of Cytotoxicity

H9c2 cells were cultured at 2 × 10^4^ cells/well in 96-well black clear bottom tissue culture plates. After 16 h, cells were treated with 50 μM tBHP, 2 mM SNP, or 5 μM Dox in the presence of 0, 1, or 3 μM Ech A for 24 h. Cytotoxicity was assessed by quantitative fluorescence assay with CellTox Green cytotoxicity assay (Promega, Madison, WI, USA). This cytotoxicity assay measures changes in membrane integrity that occur as results of cell death. They were quantified by measuring fluorescence (excitation/emission = 485 nm/530 nm) using a microplate reader (Molecular Device, Sunnyvale, CA, USA).

#### 3.2.3. Measurement of ΔΨm and ROS Level

H9c2 cells were cultured at 2 × 10^4^ cells/well in black, clear-bottom 96-well tissue culture plates. After 16 h, cells were treated with 50 μM tBHP, 2 mM SNP, or 5 μM Dox in the presence of 0, 1, or 3 μM Ech A for 1 h. Mitochondrial inner membrane potential (ΔΨm) was measured in control, tBHP-, SNP-, or Dox-treated cells in the presence of Ech A using the fluorescent dye tetramethylrhodamine ethyl ester (TMRE; excitation/emission = 549 nm/574 nm; Invitrogen, Carlsbad, CA, USA), which is sequestered by active mitochondria. Cells were stained with 200 nM TMRE for 30 min at 37 °C. After washing twice with phosphate-buffered saline (PBS), relative TMRE signal intensity in cells was analyzed using a multi-plate reader (Molecular Device, Sunnyvale, CA, USA).

Level of ROS was measured in control, tBHP-, SNP-, or Dox-treated cells in the presence of Ech A using the general ROS indicator CM-H_2_DCF-DA (excitation/emission = 492 nm/517 nm; Invitrogen, Carlsbad, CA, USA). Treated cells were incubated with 10 μM CM-H2DCF-DA for 30 min at 37 °C. After washing twice with PBS, relative CM-H_2_DCF-DA signal intensity in cells was analyzed using a multi-plate reader (Molecular Device, Sunnyvale, CA, USA).

#### 3.2.4. Measurement of Cellular and Mitochondrial ATP Level

Cellular and Mitochondrial ATP level was measured using the Mitochondrial ToxGlo assay (Promega, Madison, WI, USA) according to the manufacturer’s protocol. Briefly, H9c2 cells were cultured at 2 × 10^5^ cells/well in 60-mm tissue culture plates. After 16 h, cells were treated with 50 μM tBHP, 2 mM SNP, or 5 μM Dox in the presence of 0, 1, or 3 μM Ech A for 1 h. Harvested treated cells were resuspended by pipetting until cells were evenly dispersed. Resuspended cells were plated at 2 × 10^4^ cells/well in white, clear-bottom 96-well culture plates. Cells were separated by centrifugation at 200 g for 10 min, and 50 μL fresh medium containing 10 mM glucose (cellular ATP; glycolysis and mitochondrial ATP) or galactose (instead of glucose; only mitochondrial ATP) was added to each well. Plates were incubated at 37 °C in a humidified and CO_2_-supplemented incubator for 90 min. 100 μL assay solution was added to each well, and plates were then incubated at room temperature for 30 min. Luminescence was measured using a luminometer (Molecular Device, Sunnyvale, CA, USA).

#### 3.2.5. Measurement of OCR

OCR was measured as previously described [39]. Briefly, H9c2 cells were cultured at 2 × 10^4^ cells/well in XF24 cell culture plates (Seahorse Bioscience, Billerica, MA, USA). After 16 h, cells were treated with various doses of Ech A. After 1 h, the media was exchanged with 500 μL XF Assay Medium-modified DMEM (Seahorse Bioscience, Billerica, MA, USA) and then incubated at 37 °C without CO_2_ for 1 h. OCR was measured using an XF24 analyzer and software (Seahorse Bioscience, Billerica, MA, USA). Assay results were normalized by cell number, which was counted in each well using a Luna Automated cell counter (Logos, Annandale, VA, USA). We calculated Cellular OCR, mitochondrial OCR and coupling efficiency as follows; (1) Cellular OCR = basal OCR; without inhibitors; (2) Mitochondrial OCR = (basal OCR) − (the OCR in the presence of rotenone (complex I inhibitor) and antimycin A (complex III inhibitor)). Coupling efficiency = (basal OCR) − (the OCR in the presence of oligomycin (ATP coupler inhibitor, complex IV inhibitor)).

#### 3.2.6. Western Blot Analysis

Cell lysates were centrifuged at 14,000 rpm for 15 min at 4 °C. Protein concentrations were determined by Bradford protein assay (Bio-Rad, Hercules, CA, USA), and 30 μg of protein was loaded per lane onto 10% SDS polyacrylamide gels. Gels were transferred onto nitrocellulose membranes (Whatman, Freiburg, Germany) and incubated with specific antibodies (ERK1/2, pERK1/2, JNK, pJNK, p38, pp38, and beta-tubulin; Cell Signaling, Danvers, MA, USA). Western blotting was performed together with antibodies using a western blotting detection kit Ab signal™ (AbClon, Seoul, Korea) and detected with an LAS-3000 Plus imager (Fuji Photo Film Company, Tokyo, Japan).

### 3.3. Isolated Single Rat Cardiomyocytes

Rat hearts were mounted as previously described [40,41]. After a 15-min stabilization period in NT solution, hearts were perfused with Ca^2+^-free NT solution for 7 min, followed by Ca^2+^-free NT solution plus 0.01% collagenase (Yakult, Tokyo, Japan) for 9–13 min. Hearts were washed in oxygenated KB solution for 10 min. The atria were discarded, and the left ventricular wall and septum were cut into small pieces and agitated in KB solution to isolate cardiomyocytes.

#### 3.3.1. Measurement of Mitochondrial Inner Membrane Potential

To evaluate the protective effects of Ech A against cardiotoxic drugs in mitochondria, mitochondrial inner membrane potential (ΔΨm) was measured in control, tBHP-, SNP-, or Dox-treated isolated cardiomyocytes in the presence of Ech A using TMRE. Cells (1 × 10^6^) were stained with 200 nM TMRE (ΔΨm indicator, red color) for 30 min at 37 °C. After washing twice with PBS, cells were treated with 50 μM tBHP, 2 mM SNP, or 5 μM Dox in the presence of 0, 1, or 3 μM Ech A. After 1 h incubation at 37 °C, cells were washed with PBS, and relative TMRE signal intensity in cells was analyzed using an LSM700 confocal microscope (Carl Zeiss, Oberkochen, Germany). Acquired images were analyzed using ZEN 2009 (Carl Zeiss, Oberkochen, Germany).

#### 3.3.2. Measurement of ROS Level

Level of ROS was measured in control, tBHP-, SNP-, or Dox-treated isolated cardiomyocytes in the presence of Ech A using CM-H_2_DCF-DA. Cells were incubated with 10 μM CM-H2DCF-DA (ROS indicator, green color) for 30 min at 37 °C. After washing twice with PBS, cells were treated with 50 μM tBHP, 2 mM SNP, or 5 μM Dox in the presence of 0, 1, or 3 μM Ech A. After 1 h incubation at 37 °C, cells were washed with PBS, and relative CM-H_2_DCFDA signal intensity in cells was analyzed using an LSM700 confocal microscope (Carl Zeiss, Oberkochen, Germany). Acquired images were analyzed using ZEN 2009 (Carl Zeiss, Oberkochen, Germany).

### 3.4. Data Analysis

Unless otherwise stated, all experiments were performed in triplicate. Data are presented as mean ± standard error of the mean (SEM). Student’s *t*-tests were used to compare groups, and *P* < 0.05 was considered statistically significant.

## 4. Conclusions

These results indicate that Ech A has therapeutic potential to minimize adverse cardiotoxic effects of clinically used drugs, including SNP and Dox. We found that Ech A prevented mitochondrial dysfunction and activation of MAPK cell death signaling pathways caused by cardio/mitotoxic drug treatment. These findings could be used to develop valuable new applications of the marine drug Ech A.
